# MicroRNA-200c and microRNA-31 regulate proliferation, colony formation, migration and invasion in serous ovarian cancer

**DOI:** 10.1186/s13048-015-0186-7

**Published:** 2015-08-12

**Authors:** Fateen Farhana Ibrahim, Rahman Jamal, Saiful Effendi Syafruddin, Nurul Syakima Ab Mutalib, Sazuita Saidin, Reena Rahayu MdZin, Mohammad Manir Hossain Mollah, Norfilza Mohd Mokhtar

**Affiliations:** UKM Medical Molecular Biology Institute, Universiti Kebangsaan Malaysia, Jalan Yaa′cob Latiff, Bandar Tun Razak, 56000 Cheras, Kuala Lumpur, Malaysia; Department of Pathology, Faculty of Medicine, Universiti Kebangsaan Malaysia, Jalan Yaacob Latiff, 56000 Cheras, Kuala Lumpur, Malaysia; Department of Physiology, Faculty of Medicine, Universiti Kebangsaan Malaysia Medical Center, Jalan Yaacob Latiff, Bandar Tun Razak, 56000 Cheras, Kuala Lumpur, Malaysia

**Keywords:** MicroRNA, miR-200c, miR-31, Serous ovarian cancer, *In situ* hybridization, Migration, Invasion

## Abstract

**Background:**

Serous epithelial ovarian cancer (SEOC) is a highly metastatic disease and its progression has been implicated with microRNAs. This study aimed to identify the differentially expressed microRNAs in Malaysian patients with SEOC and examine the microRNAs functional roles in SEOC cells.

**Methods:**

Twenty-two SEOC and twenty-two normal samples were subjected to miRNA expression profiling using the locked nucleic acid (LNA) quantitative real-time PCR (qPCR). The localization of miR-200c was determined *via* LNA *in situ* hybridization (ISH). Functional analysis of miR-200c and miR-31 on cell proliferation, migration and invasion and clonogenic cell survival were assessed *in vitro*. The putative target genes of the two miRNAs were predicted by miRWalk program and expression of the target genes in SEOC cell lines was validated.

**Results:**

The miRNA expression profiling revealed thirty-eight significantly dysregulated miRNAs in SEOC compared to normal ovarian tissues. Of these, eighteen were up-regulated whilst twenty miRNAs were down-regulated. We observed chromogenic miR-200c-ISH signal predominantly in the cytoplasmic compartment of both epithelial and inflammatory cancer cells. Re-expression of miR-200c significantly increased the cell proliferation and colony formation but reduced the migration and invasion of SEOC cells. In addition, miR-200c expression was inversely proportionate with the expression of *deleted in liver cancer-1* (*DLC-1*) gene. Over-expression of miR-31 in SEOC cells resulted in decreased cell proliferation, clonogenic potential, cell migration and invasion. Meanwhile, miR-31 gain-of-function led to the down-regulation of *AF4/FMR2 family member 1* (*AFF1*) gene.

**Conclusions:**

These data suggested that miR-200c and miR-31 may play roles in the SEOC metastasis biology and could be considered as promising targets for therapeutic purposes.

**Electronic supplementary material:**

The online version of this article (doi:10.1186/s13048-015-0186-7) contains supplementary material, which is available to authorized users.

## Background

Ovarian cancer ranks as the third most common gynaecological malignancy worldwide with approximately 225,000 new cases been reported in 2011 [1]. This malignancy represents the fourth most frequently diagnosed in Malaysia and becoming a major cause of deaths in Malaysian women [2]. Epithelial ovarian cancer accounts for 90 % of all ovarian cancer and is a highly heterogeneous tumor, encompassing several histotypes with unique molecular profiles and clinical features [3]. It includes serous, endometrioid, mucinous and clear cell, with serous being the most common among all [4]. Malpica and colleagues described a novel two-tier grading system that classified SEOC into low-grade and high-grade [5] in which the latter has a higher tendency to metastasize [6].

Recently, microRNAs (miRNAs) have come into attention to better understand the molecular biology of SEOC. MiRNAs belong to a class of endogenous small non-coding RNAs family that negatively regulates mRNA at the post-transcriptional level by partially binding to the 3’ untranslated region of the target mRNA [7]. It is known that a single miRNA family could potentially target up to 500 genes and *in silico* analyses has shown that the 3' untranslated region of a single gene can be targeted by several different miRNAs [8]. To date, there are 2588 mature human miRNAs which have been deposited into the miRBase Release 21 registry [9]. As master regulators of gene expression, miRNAs can act as oncomiRs or tumor suppressor miRNAs depending on their gene targets, and alterations of miRNAs expression promote cancer development and progression [10].

MiR-200c is one of the miR-200 family members and is a marker for epithelial-mesenchymal transition (EMT) in female reproductive cancers [11]. This miRNA family is known for its role as the “guardian of the epithelial phenotype” by suppressing the EMT-inducing transcription factor, zinc finger E-box binding homeobox 1 and 2 (*ZEB1* and *ZEB2*) and in turn upregulates the expression of *E-cadherin* [12]. It has been reported that the loss of miR-200c increases the cell motility and invasiveness in breast, endometrial and ovarian cancer cell lines [11]. However, previous studies have shown that the re-expression of miR-200c reduced cell migration but did not affect *E-cadherin* expression in some cells, which suggested that miR-200c may target other genes associated with cancer metastasis [11, 13].

Although functional studies of miRNAs in ovarian cancer are steadily progressing, the knowledge on certain miRNAs for instance miR-31 is still lacking. Several studies have reported miR-31 to be upregulated in colorectal [14] and endometrial cancers [15] but downregulated in breast [16], glioma [17] and prostate cancers [18], suggesting its complexity role in cancer as it can act either as oncogenic or tumor suppressing miRNAs, depending on the origin of cancer. Since miR-31 known for its complex expression and described as a master regulator of metastasis [19], we aimed to study the roles of miR-31 in regulating SEOC.

*DLC-1* (Deleted in liver cancer-1) was initially isolated from primary hepatocellular carcinoma [20]. The gene is located on chromosome 8p21-22 and encodes a Rho GTPase-activating protein [20]. *DLC-1* is known for its tumor and metastasis suppressive role in cancer by primarily regulating the actin cytoskeleton organization, formation of actin fibres and focal adhesions [21]. The loss of *DLC-1* was not only limited to liver cancer as it has also proven to be frequently loss in multiple cancers such as breast [22], colon [23] and ovarian cancer [24]. *DLC-1* expression suppresses cell migration and invasion in colon cancer cell lines and its loss is associated with high metastatic potential [23]. However, the role of *DLC-1* in SEOC is still obscure.

Numbers of dysregulated miRNAs have been identified using different platform approaches including microarray [25–28] and sequencing [29, 30], which shows the deregulation of miRNAs could potentially serve as the diagnostic or prognostic markers specific for serous subtype of ovarian cancer. Thus, it is of great need to improve the insights of miRNA expression data that can provide promising biomarkers for SEOC or targets for therapeutic strategy. This study aimed to characterize the miRNA expression aberrations in SEOC of Malaysian patients by using qPCR on a panel of 85 cancer-related miRNAs followed by miRNA expression validation. We then performed LNA-ISH to visualize miRNA localization in the high-grade SEOC samples, subsequently expression profile in SEOC cell lines and miRNA functional analyses.

## Methods

### Clinical samples

Twenty-two SEOC with evidence of metastasis and 22 unmatched normal ovarian tissues were retrieved from the Department of Pathology, Universiti Kebangsaan Malaysia Medical Centre, Malaysia. All tissues were archived formalin-fixed, paraffin embedded (FFPE) samples from patients who were diagnosed from 2006 to 2013. The samples were histologically verified using hematoxylin and eosin staining to contain more than 70 % cancer cells by the pathologist. We obtained the unmatched normal ovarian tissues from patients who underwent total abdominal hysterectomy with bilateral salphingo-oophorectomy for benign gynecological disorders and confirmed to be free from cancer cells. Patients with secondary tumors or who underwent chemotherapy were excluded from this study. The study was approved by the UKM Medical Research Ethics Committee (Ref: UKM 1.5.3.5/244/UKM-GUP-2011-286). The clinical features were presented in Table 1.

**Table 1:** Summarized information of samples

Characteristics	Cancer samples	Control samples
	N (%)	N (%)
Age (year)		
Median age	53 [21-73]	51 [42-62]
≤50	6 (27.3 %)	10 (45.45 %)
> 50	16 (72.7 %)	12 (54.55 %)
Race		
Malay	18 (81.8 %)	10 (45.45 %)
Chinese	4 (18.2 %)	10 (45.45 %)
Others	0	2 (9.1 %)
Stage		
II	4 (18.2 %)	
III	14 (63.6 %)	
IV	4 (18.2 %)	
Grade		
Low-grade (WD)	2 (9.1 %)	
High-grade (MD or PD)	20 (90.9 %)	

WD well differentiated; MD moderately differentiated; PD poorly differentiated

### Cell lines and culture condition

Two human SEOC cell lines, CAOV3 and SKOV3 (metastatic) cells were purchased from the American Type Culture Collection (Manassas, USA). SKOV3 cells were maintained in McCoy’s 5A medium (Invitrogen, CA, USA) supplemented with 10 % fetal bovine serum (FBS) (Invitrogen). CAOV3 cells were maintained in Dulbecco's modified Eagle's medium in 4.5 g/L glucose (Invitrogen) supplemented with 10 % FBS (Invitrogen). Normal human ovarian surface epithelial (HOSE) cells were purchased from ScienCell Research Laboratories (CA, USA) and maintained in ovarian epithelial cell medium supplemented with 1 × ovarian epithelial cell growth supplement (ScienCell Research Laboratories). All cell lines were maintained at 37 °C in a humidified incubator containing 5 % CO_2_.

### Total RNA isolation and quality assessments

Total cellular RNA containing miRNAs were isolated from the FFPE samples using the High Pure miRNA Isolation kit (Roche Applied Science, Mannheim, Germany). Four 10 μm FFPE sections from each sample were pooled into a 1.5 ml tube and subjected to deparaffinization xylene at 50 °C for 5 min. The specimens were washed with absolute ethanol twice followed by protease digestion using proteinase K at 55 °C for 3 h. The RNA isolation procedure was continued according to the manufacturer’s protocol. The RNA quantity and purity were determined using the NanoDrop® ND-1000 spectrophotometer (Thermo Scientific, MA, USA) and RNA with OD260/280 ratios of 1.8–2.1. The RNA integrity was assessed with RNA Pico 6000 chip (RNA integrity number ≥ 2) and small RNA chip was used to confirm the miRNA presence in the sample using the Agilent 2100 Bioanalyzer System (Agilent Technologies, CA, USA).

### MiRNA expression profiling

To determine the differentially expressed miRNAs between the SEOC and the normal ovarian tissues, a two-step qPCR was performed using the Cancer Focus microRNA PCR panel (V1.AF) containing 85 miRNAs that have been previously reported to be related with cancer (Exiqon, Vedbaek, Denmark) (Additional file 1: Table S1). A total of 10 ng RNA was reverse transcribed to cDNA using the Universal cDNA synthesis kit (Exiqon). The reverse transcription was performed according to the manufacturer’s protocol using the Applied Biosystems Veriti™ Thermal Cycler (Applied Biosystems, CA, USA). The qPCR was then performed using the Applied Biosystems 7500 Fast Real-Time PCR System (Applied Biosystems). The run template (SDS files) was downloaded from the Exiqon’s website.

### Validation using Pick-and-Mix panel

We selected the most significantly up-regulated miRNAs in the FFPE samples following the analysis of the Cancer Focus Panel data to further validate the results. The two-step qPCR was performed using miRNA-specific primers in the Pick-&-Mix panel (Exiqon) consisting of eight miRNAs (miR-7, miR-21, miR-31, miR-182, miR-141, miR-200a, miR-200b and miR-200c) on the same set of patients and normalized to miR-27a. This assay was carried out using the Applied Biosystems 7500 Fast Real-Time PCR System (Applied Biosystems) according to the Exiqon’s protocol.

### MiRNA locked-nucleic acid (LNA) *in situ* hybridization (ISH)

Hsa-miR-200c detection probe (3′ and 5’- end labelled with digoxigenin and LNA-modified) and miRCURY LNA™ microRNA ISH Optimization Kit 2 containing miR-21 probe, U6 snRNA (both miR-21 and U6 snRNA as positive controls), scrambled miRNA LNA probe (as negative control) as well as miRNA ISH buffer were purchased from Exiqon (Vedbaek, Denmark). The ISH procedure was adapted from Nuovo with slight modifications [31].

Briefly, four μm of serial FFPE sections were mounted on the Superfrost® Plus slides (Fisher Scientific, Pennsylvania, USA). Sections were deparaffinized in fresh xylene and dehydrated in absolute ethanol for 5 min each and air dried. Then, 15 μg/ml of Proteinase K (Exiqon) was applied onto the tissue and placed in the hybridization chamber at 37 °C for 30 min. The slides were then dipped in 1× PBS for 30 s to inactivate the Proteinase K and washed with absolute ethanol. The digoxigenin (DIG)-labeled probes were denatured at 90 °C for 4 min. 2 pmol/μl of the probes diluted with 1× ISH buffer (Exiqon) was applied onto the tissue sections. Slides were placed on a hot plate at 60 °C for 5 min and into the hybridization chamber at 37 °C for 15 h. On the following day, the coverslips were carefully detached and the slides were washed in 0.2 % saline sodium citrate and 2 % bovine serum albumin solution (Sigma, Missouri, USA) at 4 °C for 10 min. The chromogenic detection of the miRNA LNA-ISH probe was performed with anti-DIG-alkaline phosphatase conjugate (1:200 dilution of the conjugate in blocking reagent) and incubated at 37 °C for 30 min. The miRNA expression was detected by 4-nitro-blue-tetrazolium and 5-bromo-4-chloro-3-indolynitrolphosphate substrate (Roche Applied Science). The slides were counterstained with nuclear fast red (Roche Applied Science). For image acquisition, the Nikon Eclipse 80i microscope (Nikon, Melville, USA) integrated with the Image-Pro Express 6.0 software (Media Cybernetics, Silver Spring, USA) was utilized. The sample images were captured with 200× magnification.

### *In silico* miRNA target prediction analysis

To determine the miRNA-putative gene targets, we used the comprehensive miRNA target prediction algorithm, miRWalk, from the publicly available website, www.umm.uni-heidelberg.de/apps/zmf/mirwalk/(March 2011 release). The database integrates ten miRNA target prediction algorithms (including miRWalk itself). The miRNA-mRNA interactions which are predicted by at least five algorithms out of 10 algorithms were retained. Data mining from the cancer microarray database, Oncomine (https://www.oncomine.org/), was utilized to identify the candidate down-regulated genes (fold-change < −2, *p* < 0.05) that involve in ovarian carcinogenesis. The potential target gene list was narrowed down by selecting cancer-related genes and pathways using the DAVID Bioinformatics Resources v6.7 tool (https://david.ncifcrf.gov) which is linked with the Kyoto Encyclopedia of Genes and Genomes (KEGG) pathway annotation. The gene list generated from the pathway enrichment analysis was used to overlap with the putative miRNA targets from miRWalk.

### Transient transfection of mature miRNAs

Cells (5 × 10^5^) were seeded into each well of a 6-well plate and cultured in antibiotic-free culture media without serum until reached 70 % confluency. Mature miRNA molecules were transiently transfected into SEOC cell lines using Lipofectamine 2000 (Invitrogen) in Opti-MEM® I reduced serum medium (Invitrogen) at 150 nM concentration. For gain-of-function experiments, we used miRIDIAN miRNA mimics hsa-miR-200c-3p and hsa-miR-31-5p together with scrambled miRNA mimic negative control (GE Healthcare Dharmacon, Inc., Lafayette, CO, USA). While for loss-of-function experiments, miRCURY LNA miRNA inhibitors miR-200c-3p and miR-31-5p (Exiqon) with scrambled miRNA inhibitor control (Exiqon) were used. 5’ terminal fluorescein-labeled scrambled miRNA inhibitor control was employed to assess the transfection efficiency using the BD FACSAria II flow cytometer (BD Biosciences, San Jose, USA).

### Gene expression analysis of miRNA-transfected SEOC cells

Total RNA was isolated from the miRNA-transfected cells using the QIAzol lysis reagent and miRNeasy Mini Kit (Qiagen, CA, USA) according to the manufacturer’s protocol. Two μg of RNA template was converted to cDNA using the High Capacity RNA-to-cDNA kit (Applied Biosystems). QPCR analysis was carried out for miR-200c and miR-31 target genes, *DLC-1* and *AFF1*, respectively using the TaqMan® gene expression assay. The expression values of the genes of interest in each of the transfected cells were normalized to the expression value of the endogenous control, *GAPDH*. Relative fold change of the targeted gene levels between the transfection groups were determined by the 2^-∆∆CT^ method [32]. All experiment reactions were performed in triplicates.

### Cell proliferation assay

The SEOC cells (1 × 10^4^) were seeded and cultured overnight in a 96-well plate. On the following day, the cells were transiently transfected with 150 nM miRNA mimics, inhibitors and respective NC. The proliferation rate was determined by PrestoBlue™ Cell Viability reagent (Invitrogen) at 48 h post-transfection according to the manufacturer’s protocol. The fluorescence was measured using a microplate reader SkanIt RE for Varioskan Flash 2.4 (Thermo Fisher Scientific, Massachusetts, USA) at excitation/emission wavelengths of 560/590 nm. Experiment was performed in six replicates.

### Colony formation assay

After 24 h transfection with 150 nM miR-200c and miR-31 mimics, inhibitors and respective NC, 500 cells were plated in a 6-well plate with complete media and the plate was swirled to ensure an even distribution of the cell. The cells were grown in 37 °C incubator with 5 % CO_2_ for 10 days with media replacement every 3 days. At day 10, the media was removed and cells were washed twice with PBS. The colonies were fixed with 50 % cold methanol for 10 min, dried and stained with 0.5 % crystal violet solution for 30 min. In order to remove the excess staining, the plate was washed three times with tap water. Images of the stained plates were captured, and the cell colonies containing more than 50 cells were counted. Each treatment was performed in triplicates.

### Transwell cell invasion and migration assay

The QCM™ 24-well Fluorimetric Cell Invasion assay kit (ECM554; Chemicon) and the Migration kit (ECM509; Chemicon) were used to assess the migration and invasion properties of the miRNA-transfected SEOC cells. The assays were carried out according to the manufacturer’s protocol. Both kits used an insert polycarbonate membrane with an 8 μm pore size. The insert in the invasion kit was pre-coated with a thin layer of ECMatrix™ which occluded the membrane pores and blocked the migration of non-invasive cells. After 48 h transfection, cells were harvested and re-suspended in a serum-free medium. The upper chamber was filled with 300 μL cell suspension in triplicate in a 24-well plate, and 500 μl of complete medium was added to the bottom well. Following 24 h incubation, the cells that had not migrated were pipetted out from the upper surface of the inserts, and the cells that had migrated to the lower filters were detached using the cell detachment solution. Images of three random fields (×40 magnification) were captured from each well. The migrated and invaded cells were lysed and the relative invasion was determined by fluorescence with 480/520 nm filter set. The miRNA-transfected cells were normalized to their respective scrambled negative control groups.

## Statistical analysis

### MiRNA expression profiling

QPCR data obtained from FFPE samples were analyzed to identify the common miRNAs that are present in SEOC. The C_T_ values obtained from the expression profiling were imported into the Exiqon GenEx Version 5.4.2 software in .txt format. Pre-processing was done prior to data analysis including (1) inter-plate calibration, (2) selecting reference genes using the NormFinder and (3) normalization against the endogenous control miRNA, which is miR-27a. For the quality control step, principal component analysis (PCA) plot was generated. To determine the significant differentially expressed miRNAs, robust statistical analysis using the Kruskal-Wallis and LIMMA tests were carried out separately using a p-value < 0.05 and log_2_ fold-change between ≤ −1.0 and ≥ 1.0. The overlapping miRNAs found using both tests were subjected for further analysis. Outliers were identified by the data points that fall beyond UQ + 1.5.IQD or LQ-1.5.IQD in the box plot (UQ: upper quartile, IQD: inter-quartile distance, LQ: lower quartile). UQ and LQ are the 75^th^ and 25^th^ percentiles respectively. Outliers were then replaced with the group median. Heatmap analysis and hierarchical clustering were generated using the R programming software for miRNA expression pattern visualization. To further confirm the direction of miRNAs expression, the significant miRNAs were subjected to experimental validation.

### MiRNA functional analysis

For miRNA functional analysis, all statistical analyses were performed using GraphPad Prism 6.0 statistical software. Experimental data was presented as mean ± standard deviation (S.D.). The differences between groups were analyzed using Student’s *t*-test with a *p* < 0.05 was regarded as statistically significant. All experiments were conducted in triplicates to ensure reproducibility of the results.

## Results

### Differentially expressed miRNAs in metastatic SEOC versus normal ovary

LNA™-qPCR was performed in order to determine the dysregulated miRNAs in SEOC and normal ovarian tissues. We obtained a total of 22 FFPE tissues from the patients who had been diagnozed with SEOC that did not receive chemotherapy. All of the samples had metastatic evidence within the peritoneal cavity. Eighteen of the patients were Malays and four were Chinese. There was no SEOC FFPE sample from Indian or other races included in this study. According to the FIGO classification, four samples were in Stage II, 14 were in stage III and four were from the Stage IV. Based on Malpica’s 2-tier grading, two of the patients were diagnosed with low-grade SEOC and 20 of them were high-grade SEOC. The clinicopathological data of the 22 patients of SEOC with metastasis evidence and 22 normal controls used in this study are shown in Table 1.

**Fig. 1:**
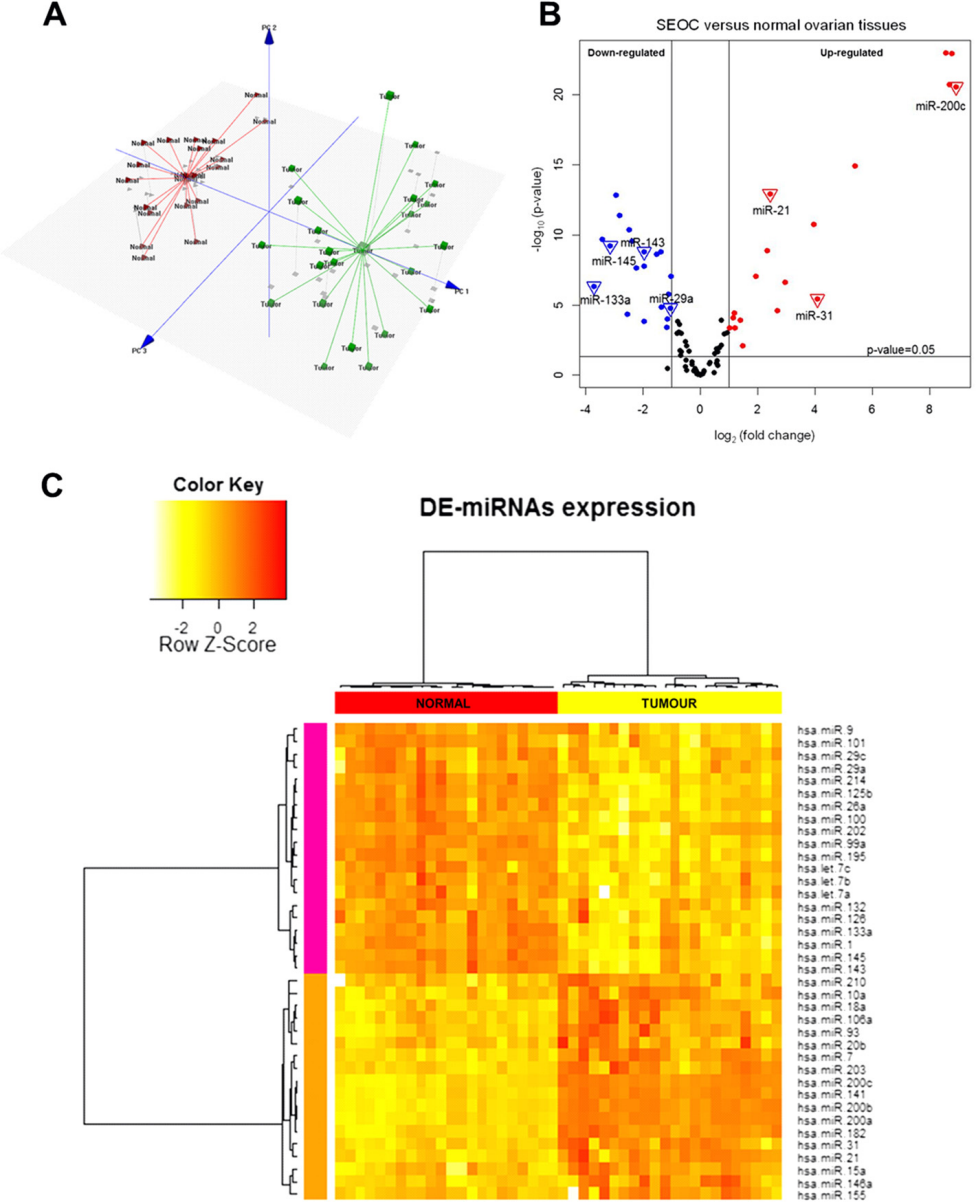
MicroRNA (miRNA) expression profiling of SEOC and normal ovarian tissues. **a** The principal component analysis (PCA) plot generated a clear segregation between SEOC (green) and normal ovarian tissues (red). **b** The volcano plot representing significance of identified miRNAs which are differentially expressed with *p* < 0.05. The up-regulated miRNAs are shown in red while down-regulated miRNAs are shown in blue. **c** Hierarchical clustering of the 38 differentially expressed (DE) miRNAs. Samples are designated along the vertical axis and clustered by the colour bar in between the dendrogram and heatmap. Red denotes normal ovary group while yellow denotes SEOC group. The colour key illustrates the miRNAs relative expression across all samples. Red illustrates higher expression level than the mean and yellow illustrates lower expression level than the mean

Prior to data analysis, the endogenous miRNA candidate that would serve as normalization control was determined using the NormFinder software. MiR-27a was found to be the most stably expressed miRNA in all samples by having the lowest S.D. value which indicates the miRNA is a good endogenous control for data normalization. Thus, miR-27a was used to normalize the miRNAs expression in both SEOC and normal ovarian tissues. Out of 85 miRNAs analysed in the qPCR cancer panel, 38 miRNAs was significantly dysregulated with a log_2_ fold-change of either ≤ −1.0 or ≥ 1.0 with *p* < 0.05, of which 18 miRNAs were up- and 20 were down-regulated in SEOC compared to the normal samples (Tables 2 and 3). The PCA plot clearly segregated the samples into two distinct groups; SEOC and normal ovarian samples (Fig. 1a). The volcano plot illustrated the relationship between the –log_10_ p-value and the log_2_ fold change in SEOC versus normal ovary samples (Fig. 1b). A representative hierarchical clustering based on Euclidean algorithm was generated with the heatmap where two distinctive expression profiles were observed (Fig. 1c). The complete list of the 85 miRNAs evaluated across all the samples in this study is summarized in Additional file 2: Table S2.

**Table 2:** MicroRNAs significantly up-regulated in metastatic SEOC compared to normal ovarian tissues

miRID	Adjusted p-value (Kruskal-Wallis)	Adjusted p-value (LIMMA)	log_2_ fold change	Chromosomal location	Cytogenetic band
Up-regulated					
hsa-miR-200c	1.91E-07	2.64E-21	8.921469061	12: 6963699-6963766 [+]	12p13.31
hsa-miR-141	1.91E-07	1.12E-23	8.787092856	12: 6964097-6964191 [+]	12p13.31
hsa-miR-200b	1.91E-07	1.86E-21	8.699603684	1: 1167104-1167198 [+]	1p36.33
hsa-miR-200a	1.91E-07	1.06E-23	8.562872035	1: 1167863-1167952 [+]	1p36.33
hsa-miR-182	1.91E-07	1.13E-15	5.411527936	7: 129770383-129770492 [-]	7q32.2
hsa-miR-31	6.93E-05	3.77E-06	4.094320534	9: 21512115-21512185 [-]	9p21.3
hsa-miR-7	5.82E-07	1.72E-11	3.971773447	9: 83969748-83969857 [-]	9q21.32
hsa-miR-203	2.23E-05	2.36E-07	2.966799585	14: 104117405-104117514 [+]	14q32.33
hsa-miR-10a	0.000681963	2.62E-05	2.701327257	17: 48579838-48579947 [-]	17q21.32
hsa-miR-21	1.91E-07	1.23E-13	2.431890348	17: 59841266-59841337 [+]	17q23.1
hsa-miR-18a	1.95E-07	1.33E-09	2.329697689	13: 91350751-91350821 [+]	13q31.3
hsa-miR-93	1.91E-06	8.84E-08	1.947479146	7: 100093768-100093847 [-]	7q22.1
hsa-miR-20b	0.049868623	0.008417024	1.489542936	X: 134169809-134169877 [-]	Xq26.2
hsa-miR-146a	0.002542269	0.000122875	1.38901606	5: 160485352-160485450 [+]	5q33.3
hsa-miR-155	0.000725017	0.000447306	1.209141398	21: 25573980-25574044 [+]	21q21.3
hsa-miR-15a	9.13E-05	3.75E-05	1.192194165	13: 50049119-50049201 [-]	13q14.2
hsa-miR-106a	9.77E-05	7.99E-05	1.140778656	X: 134170198-134170278 [-]	Xq26.2
hsa-miR-210	0.002542269	0.000443277	1.015069322	11: 568089-568198 [-]	11p15.5

**Table 3:** MicroRNAs significantly down-regulated in metastatic serous ovarian cancer compared to normal ovarian tissues

miRID	Adjusted p-value (Kruskal-Wallis)	Adjusted p-value (LIMMA)	log_2_ fold change	Chromosomal location	Cytogenetic band
Down-regulated					
hsa-let-7a	1.18E-05	8.84E-08	−1.029	9: 94175957-94176036 [+]	9q22.32
hsa-miR-29a	0.000115739	1.75E-05	−1.032	7: 130876747-130876810 [-]	7q32.3
hsa-miR-126	9.83E-06	1.70E-06	−1.107	9: 136670602-136670686 [+]	9q34.3
hsa-miR-29c	0.000265228	9.82E-05	−1.148	1: 207801852-207801939 [-]	1q32.2
hsa-miR-132	0.001852309	0.000402718	−1.162	17: 2049908-2050008 [-]	17p13.3
hsa-miR-101	5.45E-05	1.42E-05	−1.352	1: 65058434-65058508 [-]	1p31.3
hsa-miR-26a	2.74E-07	1.71E-09	−1.373	3: 37969404-37969480 [+]	3p22.2
hsa-let-7b	8.28E-07	2.42E-09	−1.509	22: 46113686-46113768 [+]	22q13.31
hsa-miR-143	2.06E-06	1.58E-09	−1.953	5: 149428918-149429023 [+]	5q32
hsa-miR-9	0.000532847	0.000152253	−1.955	1: 156420341-156420429 [-]	1q22
hsa-let-7c	2.21E-06	1.72E-08	−1.966	21: 16539828-16539911 [+]	21q21.1
hsa-miR-214	2.06E-06	2.30E-08	−2.233	1: 172138798-172138907 [-]	1q24.3
hsa-miR-100	2.63E-07	2.73E-10	−2.369	11: 122152229-122152308 [-]	11q24.1
hsa-miR-125b	2.87E-07	4.47E-11	−2.488	11: 122099757-122099844 [-]	11q24.1
hsa-miR-202	6.46E-05	4.59E-05	−2.533	10: 133247511-133247620 [-]	10q26.3
hsa-miR-99a	1.91E-07	4.00E-12	−2.821	21: 16539089-16539169 [+]	21q21.1
hsa-miR-195	1.91E-07	1.46E-13	−2.945	17: 7017615-7017701 [-]	17p13.1
hsa-miR-145	7.96E-07	6.22E-10	−3.137	5: 149430646-149430733 [+]	5q32
hsa-miR-1	2.06E-06	2.04E-10	−3.426	20: 62554306-62554376 [+]	20q13.33
hsa-miR-133a	4.12E-05	4.92E-07	−3.705	18: 21825698-21825785 [-]	20q13.33

miRID: miRNA identifier from miRBase Release 21

### The Pick-&-Mix qPCR validation of selected miRNAs

**Fig. 2:**
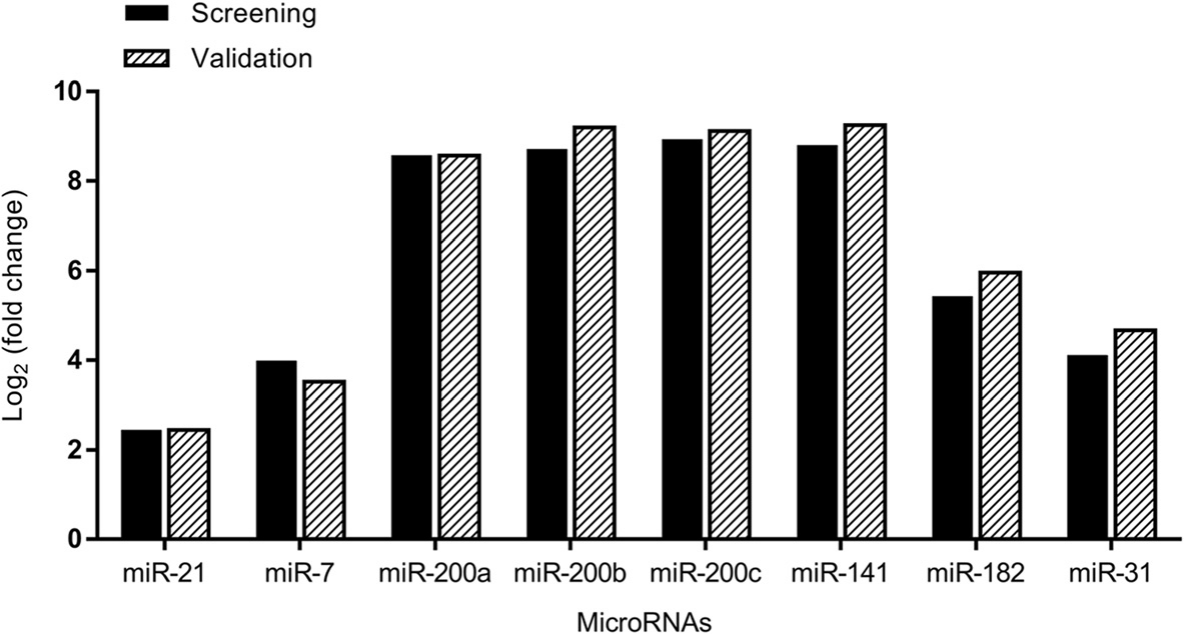
QPCR validation of selected miRNAs confirmed the miRNA profiling results. The log_2_ fold-change is shown in both screening and validation analysis for SEOC versus normal ovary with *p* < 0.05. The black bars represent values from screening data and bars with diagonal pattern represent validation data

QPCR validation results of the eight selected miRNAs (miR-7, miR-21, miR-31, miR-182, miR-141, miR-200a, miR-200b and miR-200c) showed concordance with the qPCR analysis. These findings confirmed the miRNA expression profiling results as shown in Fig. 2.

### MiR-200c predominantly localized in the cancer epithelia

**Fig. 3:**
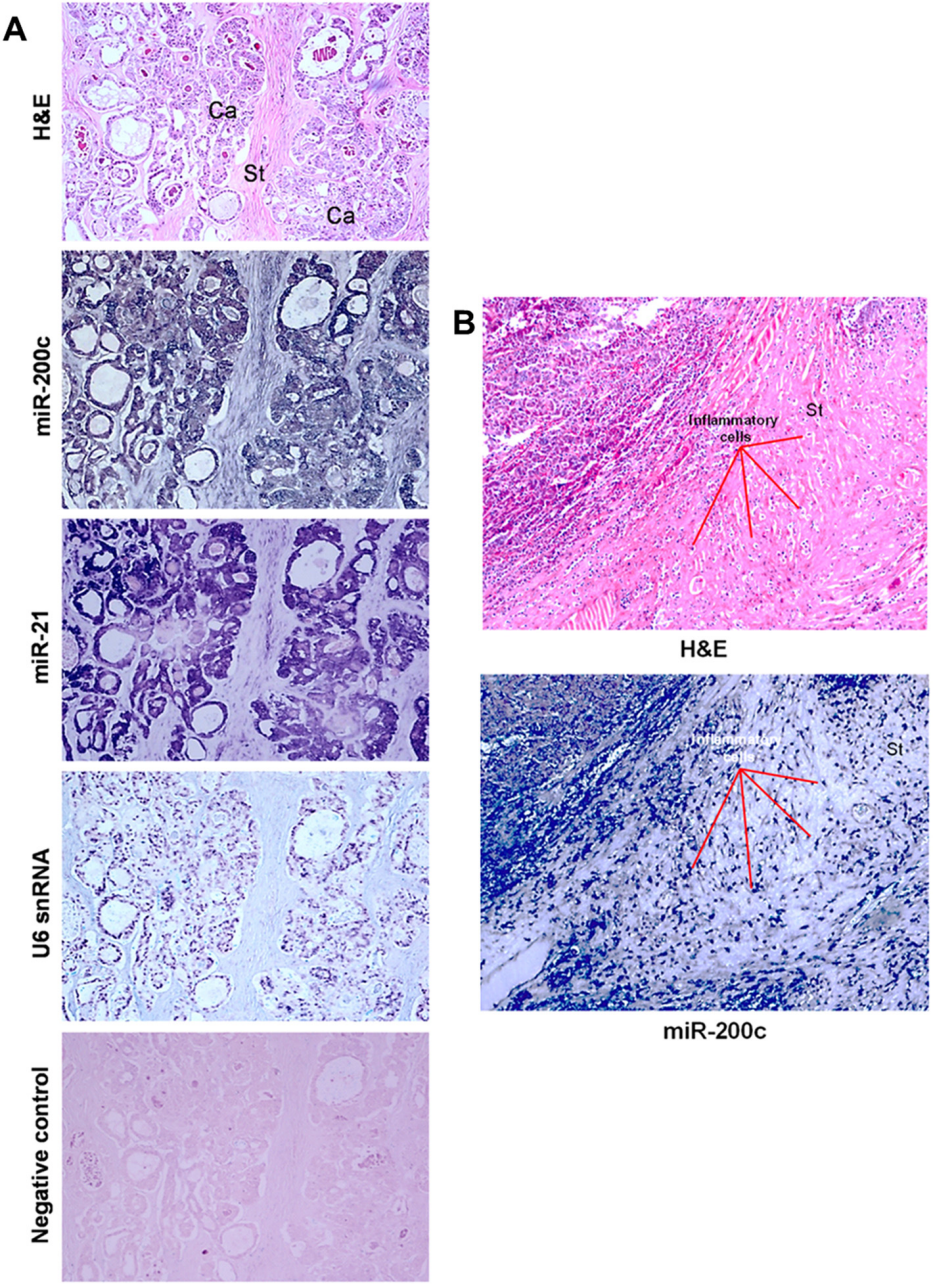
MiR-200c localized in the epithelial cancer cell and inflammatory cells but absent in stroma. **a** The tissue was stained with H&E to identify the cancer cellular structures. Intense LNA-miR-200c ISH signal is detected in the cytoplasmic region, predominantly in the epithelial of cancer cells. Weak miR-200c ISH signal is seen in the stromal compartment. MiR-21 (positive control) exhibits the same pattern of localization as miR-200c. The U6 ISH signal is solely in the nuclei of all cell types. There was no ISH signal detected in the negative control. Ca stands for cancer cells and St stands for stroma. **b** Localization of miR-200c was also detected in the inflammatory cells of SEOC. All images were captured under 200× magnifications

The up-regulation and localization of miR-200c in SEOC were further validated *via* ISH assay upon the intact SEOC FFPE tissue. MiR-200c probe gave an intense bluish ISH signal was readily detectable in the cytoplasm of the epithelial cancer cells without background staining. Whereas, a weak miR-200c signal in the adjacent stromal compartment was observed (Fig. 3a). MiR-200c also displayed a dense blue chromogenic nucleoli staining in the SEOC case (Additional file 3: Figure S1). The miR-21 ISH signal which act as another positive control was also observed in the cytoplasmic compartment of the cancer cells. The ISH signal for the U6 snRNA was exclusively expressed in the nuclei of all cell types. In addition, there was no specific ISH signal detected when applying scrambled sequence probe which further confirmed the probes’ specificity in detecting their respective miRNAs. We also detected inflammatory cells showed strong ISH signal for miR-200c (Fig. 3b).

### *In silico* analyses reveal the putative gene targets and pathways of miRNAs

Given that a miRNA has the potential to target a large number of genes, we employed a publicly available database, miRWalk that consists of ten different prediction algorithms, thus avoiding the false positive miRNA target predictions. After integrating the Oncomine data (Adib et al. 2004) with the results from the miRWalk *in silico* analysis, we identified that *DLC-1* which was predicted by eight target prediction algorithms as the potential target of miR-200C. We employed similar strategies for miR-31 and identified *AFF1* as the putative target for this miRNA, predicted by six prediction algorithms. The canonical pathways that may be regulated by the miRNAs were also evaluated. The predicted cancer-related pathways are summarized in Additional file 3: Table S3 for miR-200c and Additional file 3: Table S4 for miR-31. It is worth stating that p53, MAPK and Wnt signalling pathways were some of the pathways shared by both miRNAs, suggesting the possible roles of these miRNAs upon the mentioned pathways in resulting SEOC metastasis.

### Quantitative analysis of miR-200c and miR-31 in SEOC cell lines

**Fig. 4:**
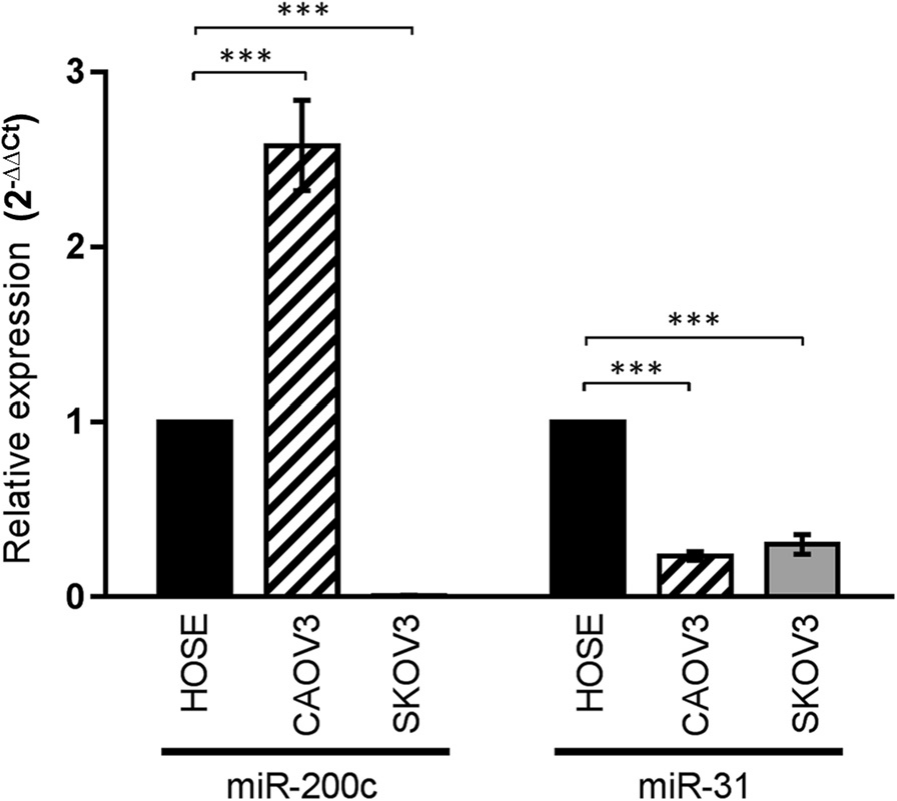
Expression status of endogenous miR-200c and miR-31 levels in SEOC cell lines compared to human ovarian surface epithelial (HOSE) cells. The miR-200c level is significantly up-regulated in CAOV3 cells but extremely down-regulated in SKOV3 cells. Meanwhile, miR-31 level was significantly down-regulated both in CAOV3 and SKOV3 cells Statistical analysis was performed using Student’s *t*-test. Error bars represent standard deviation (S.D.). (***p < 0.001)

We examined the miR-200c and miR-31 expression in two SEOC cell lines; CAOV3 (primary tumor SEOC cell) and SKOV3 (isolated from ascites) and in non-tumorigenic cell (HOSE) using qPCR. As presented in Fig. 4, CAOV3 cell expressed a higher level of miR-200c than HOSE whereas SKOV3 expressed lower level of miR-200c than HOSE. Meanwhile, the expression level of miR-31 was down-regulated in both CAOV3 and SKOV3 cells relative to the HOSE cells (*p* < 0.001), which surprisingly found to be contradictory to the expression level that we observed in the cancer tissue samples (Additional file 3: Figure S2). Despite the discrepancy of miR-31 expression found in cancer tissues and cultured cell lines, we next determine whether the modulations of these miR-200c and miR-31 would have any effect on the biological behaviours of these two SEOC cell lines.

### Re-expression of miR-200c inhibited the mRNA *DLC-1* level, enhanced proliferation and clonogenic potential but suppressed invasion and migration of SEOC cells

**Fig. 5:**
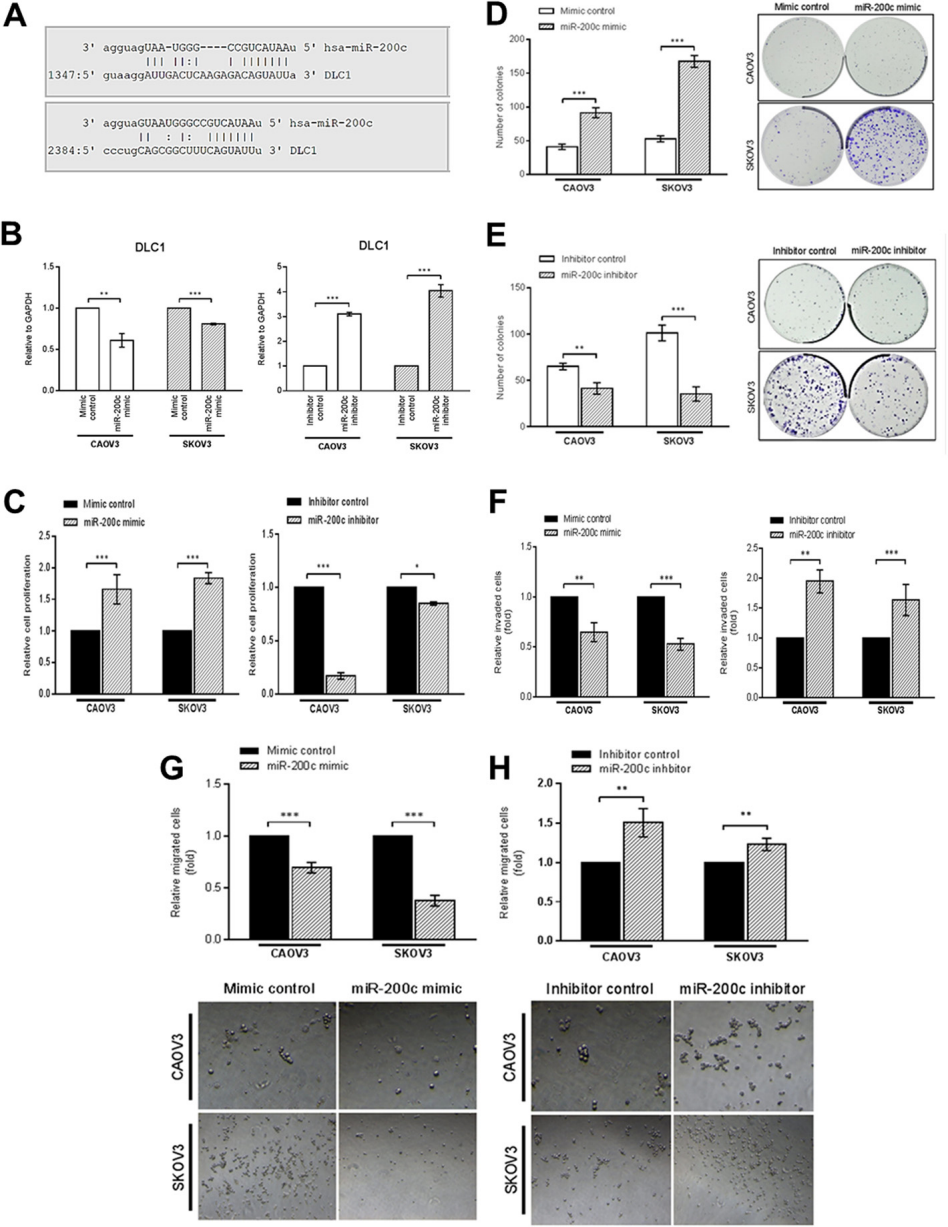
MiR-200c down-regulates *DLC-1*, promotes proliferation and colony formation but suppresses SEOC cells invasion and migration. **a** The alignment of nucleotide sequence between the miR-200c and complementary sequence on *DLC-1* 3’untranslated region (UTR) interactions. The colon (:) symbol denotes wobble base pair between guanine (G) and uracil (U) nucleotides. **b** QPCR showing down-regulated *DLC-1* mRNA following transient transfection of miR-200c mimic and inhibitor in SEOC cells. **c** The miR-200c mimic transfection promoted cell proliferation and inhibition of miR-200c reduced cell proliferation. **d** Up-regulation of miR-200c enhanced colony formation but **(e)** down-regulation of miR-200c caused colony formation suppression. **f** miR-200c over-expression increased cell invasion whereas miR-200c down-regulation reduced cell invasion. **g** MiR-200c inhibited cell migration in SEOC cells and **(h)** The inhibition of miR-200c induced cell migration. All experiments were conducted in triplicates. Data are presented as mean with error bars representing the S.D. Statistical analysis of the microRNA functional studies were done using *t*-test. (*p < 0.05, **p < 0.01, ***p < 0.001)

We over-expressed or inhibit miR-200c in the SEOC cells and examined changes in putative target mRNA, cell proliferation, colony formation ability, cell invasion and migration. Transfection efficiency was determined by a flow-cytometric analysis of transfected exogenous miRNA labeled with fluorescein (Additional file 3: Figure S3). The 3’ UTR of *DLC-1* contains two putative binding sites for miR-200c (Fig. 5a). Since the *DLC-1* level was down-regulated in SEOC, we hypothesized that there would be significant changes in the expression level of *DLC-1* upon transfecting the cells with either miR-200c mimic or inhibitors. Total RNA was purified from both CAOV3 and SKOV3 at 48 h post-transfection with miR-200c mimics and inhibitors, and *DLC-1* expression level was quantified by using the qPCR. Consistent with our hypothesis, we discovered that over-expression of miR-200c reduced the *DLC-1* expression whereas the inhibition of miR-200c resulted in up-regulation of *DLC-1* expression (Fig. 5b).

As depicted in Fig. 5c, induced expression (gain-of-function) of mature miR-200c led to significant increase in SEOC cell proliferation compared to the negative control. Meanwhile, the delivery of miR-200c inhibitor into the SEOC cell lines significantly reduced the cell proliferation. Similar observation was seen in the colony formation assay whereby the over-expression of miR-200c promoted the clonogenic potential (Fig. 5d) and inhibition of miR-200c caused suppression in the clonogenic potential as relative to the negative controls in both cancer cell lines (Fig. 5e). Next, we observed that over-expression of miR-200c reduced the invasion of SEOC cells through the ECMatrix™, whereas the inhibition of miR-200c promoted the invasiveness of the SEOC cells (Fig. 5f). Likewise, the migration of cells through the filter membrane was significantly repressed in response to miR-200c mimic treatment (Fig. 5g), whilst inhibition of miR-200c exhibited the opposite effects (Fig. 5h).

### Over-expression of miR-31 down-regulated *AFF1*, suppressed cell proliferation, clonogenic potential, migration and invasion properties of SEOC cells

**Fig. 6:**
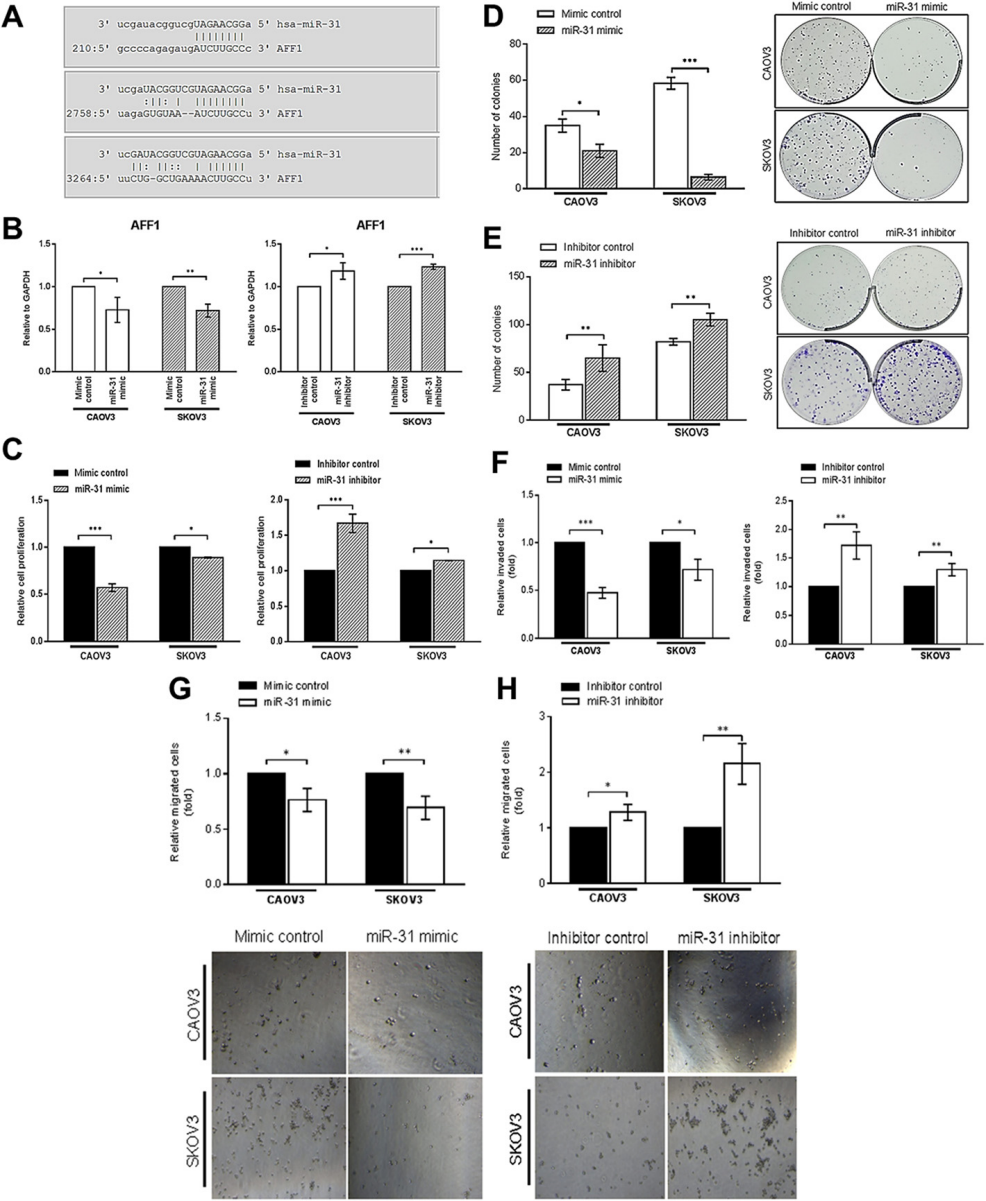
MiR-31 down-regulates *AFF1,* reduces SEOC cell proliferation, colony formation, invasion and migration. **a** The three predicted locations of miR-31 target binding sites on *AFF1* 3’UTR. The vertical lines denote the “seed” regions by Watson-Crick base pairing. The colon (:) symbol denotes wobble base pair between guanine (G) and uracil (U) nucleotides. **b** The *AFF1* expression was negatively regulated by miR-31 when measured with qPCR. **c** MiR-31 significantly reduced cells proliferation and inhibiting miR-31 markedly induce proliferation of SEOC cell lines. **d** Introduction of exogenous miR-31 into SEOC cells significantly inhibited clonogenic potential while **(e)** inhibition of miR-31 exhibits the opposite effect. Representative images of crystal violet stained colonies in each treatment were shown. **f** The cell invasion was assessed using pre-coated ECMatrix™ transwell membranes. **g** The cell migration assay using transwell membrane showed SEOC-miR-31 transfected cells were significantly less than that of SEOC-negative controls transfected cells. **(H)** Inhibition of miR-31 reversed the effects and enhanced SEOC cells migration. (*p < 0.05, **p < 0.01, ***p < 0.001, *t*-test)

There are three predicted binding sites for miR-31 at the 3’ UTR of *AFF1* gene (Fig. 6a). We found that *AFF1* mRNA level was negatively regulated by miR-31 (Fig. 6b). As shown in Fig. 6c, the cell proliferation property of the CAOV3 and SKOV3 cells transfected with miR-31 mimic was significantly inhibited while inhibition of miR-31 further enhanced the SEOC cells proliferation. Similar results were observed for the colony formation assay in which miR-31 over-expression in SEOC cell lines markedly reduced the cells ability to grow and form colony (Fig. 6d), whereby the knockdown of endogenous miR-31 increased the cell survival to form colonies (Fig. 6e). In addition, up-regulation of miR-31 showed a significant reduction in the cells invasion and vice-versa when treated with miR-31 inhibitor (Fig. 6 f). For the migration assay, miR-31-transfectants had their migration property reduced (Fig. 6g). In contrast, the cells migration increased upon treatment with miR-31 inhibitor (Fig. 6h).

## Discussion

SEOC exhibits as the most common histological types of ovarian cancer and patients often succumbed to the metastatic disease. Generally, ovarian cancer patients with different histological subtypes received a standard, non-personalized treatment. Therefore, search for potential biomarkers may help in the development of a better histological-specific treatment, hence improve patients’ outcome. Most of the cancer miRNA-based biomarkers that have been reported thus far were from Western populations. However, data on miRNA profile of SEOC patient in the Malaysian population has not been reported. Here, we characterized miRNA expression profiling on a panel of 85 cancer-related miRNAs in twenty-two unmatched pairs of SEOC samples. The inclusion of only serous subtype tissue within the cancer group in our study has reduced the variations that were caused by tumor type heterogeneity and may lead to the identification of serous-specific biomarker.

We identified a total of 18 up-regulated and 20 down-regulated miRNAs in SEOC versus normal ovarian samples. The miR-200 family that includes miR-141, miR-200a, miR-200b and miR-200c were the most up-regulated miRNAs in our samples. These highly expressed miRNAs showed relatively good concordance with previous studies on SEOC [25–30]. Kan and colleagues have shown this miRNAs family was also elevated in the serum samples of SEOC patients [33]. We also found the same pattern of miR-100 and miR-125b under-expression in our samples with three other studies [26, 28, 30]. We could conclude that the miR-200 family, miR-100 and miR-125b remain the most consistently dysregulated in SEOC and may potentially be used as the diagnostic tool for future therapy. The high expression level of miR-200c measured by qPCR was verified by the chromogenic LNA-ISH analysis and it is evident that the miRNA were being expressed and localized in the cytoplasm of epithelial cancer cells. We also observed miR-200c ISH signal in the inflammatory cells population which may suggest the regulation of miR-200c on inflammation component and consequently contribute to the invasiveness of SEOC.

Cumulative evidence has demonstrated the complex expression and role of miR-200c in SEOC. Hence, we used the *in vitro* model to understand the miR-200c expression in different stages of SEOC by using HOSE as the normal ovarian cell, CAOV3 cell originated from primary SEOC site and SKOV3 cell originated from ascites of SEOC patient. Interestingly, we found that miR-200c expression was significantly higher in the CAOV3 cells but dramatically down-regulated in SKOV3 cells. Our *in vitro* data showed that the down-regulation of miR-200c causes up-regulation of the tumor suppressor gene, *DLC-1.* In other studies, the restoration of *DLC-1* gene could effectively arrest the proliferation of ovarian cancer cells [34] and inhibit migration and invasion in breast cancer cells [35]. However, we observed that the re-expression of miR-200c in the SEOC cell lines stimulated cell proliferation and colonies formation but represses cell migration and invasion. These observations were consistent with previous studies investigated in other cancer types [36, 37]. They have demonstrated that miR-200c regulates the epithelial-mesenchymal transition by targeting *ZEB1* and *ZEB2*, resulting in increased expression of the cell-cell adhesion molecule, *E-cadherin* and eventually causes re-epithelialization of cells to form metastatic foci at secondary site. Our present data demonstrates that miR-200c could suppress invasion and migration in SEOC cells and might add to the evidences that miRNAs could serve as potential targets for suppressing tumor metastasis.

Previously, Ren et al. has demonstrated that the expression of endogenous DLC-1 protein were low relative to the expressions in their normal counterpart [24]. The low expression of *DLC-1* was significantly associated with advanced FIGO stage, ascites and lymph node metastasis [24]. Another study using the gene transfection approach has demonstrated that the tumor suppressive activity of *DLC-1.* The restoration and upregulation of *DLC-1* expression could dramatically repress the proliferative potential of ovarian cancer cells [34]. Meanwhile, the reintroduction of *DLC-1* in metastatic breast cancer cell line leads to reduction of migration and invasion properties in both *in vitro* and *in vivo* models [35]. This experiment confirms that DLC-1 is not only limited to tumorigenesis but also in metastasis. An effort linking between miRNAs function, *DLC-1* expression and metastatic properties was recently reported by Pacurari et al. in metastatic non-small cell lung cancer cells [13]. They have shown that the re-expression of miR-200c in the metastatic cells could reduce DLC-1 protein level. Although we showed that miR-200c could reduce the *DLC-1* mRNA level in SEOC cells, there is also possibility that *DLC-1* might not be a direct target of miR-200c or be activated by other miRNAs whom their regulatory activities are associated with miR-200c expression. Therefore, the mechanisms on how the interaction between miR-200c and *DLC-1* in regulating SEOC clonogenic potential and invasiveness need in-depth explorations.

MiR-31 expression has been reported to be frequently altered in different malignancies and plays a significant role in metastasis process. The functional role of this miRNA is rather complicated as it can act as either tumour-suppressive or oncogenic, depending on the cellular context. MiR-31 expression was elevated in endometrial cancer [15] but paradoxically down-regulated in breast [16] and prostate cancer [18]. We noted that miR-31 expression was elevated in our local samples. However, contrary to our data, miR-31 was found down-regulated in previous studies [29, 30]. We also observed that miR-31 was down-regulated in the SEOC cell lines which was isolated from Caucasian SEOC patients. Several causes may contribute to the discrepancy of the miR-31 expression including the different applications of technologies in profiling miRNAs expression as previous studies have used the deep sequencing approach. Besides, the selection of normal ovarian as the control group may also be the contributing factor in the disagreement as previously, primary cultured HOSE cells were utilized as the control [29, 30]. Zorn et al. had shown that the primary HOSE cells had a significant low correlation of gene expression with ovarian tissue and ovarian surface epithelial brushings [38]. The exposure of cells to tissue culture conditions significantly altered gene expression by directly disturbing transcriptional regulation [38]. Thus, our present approach comparing the results from cancer tissues to normal ovarian tissue is more relevant than the previous study that used cultured HOSE cells as the control group.

It was reported that miR-31 expression could influence numerous cancer-related phenotypes including cell proliferation and growth, migration and metastasis. Hence, it is of great interest to study the biological function of miR-31 in SEOC. We used exogenous miR-31 mimic to transiently over-express miR-31 *in vitro* and observed that the cell proliferation and colony forming ability of CAOV3 and SKOV3 were inhibited as compared to cells transfected with negative control. The suppression of cell proliferation and colony formation in both of the cells indicates the significance role of miR-31 in SEOC survival. Our *in vitro* miRNA functional analyses also revealed the role of miR-31 in suppressing the key steps of tumor metastasis, in which the migration and invasion of SEOC cells were suppressed by miR-31 compared with the negative control. In breast cancer, the re-expression of miR-31 caused the reduction of metastasis [38], which shows that miR-31 most likely play similar roles in SEOC just as breast cancer. Similar to another study in glioblastoma, miR-31 was shown to suppress the migration and invasion of glioma cells by directly targeting the cytoskeletal protein, radixin [17]. Hence, it further proves that miR-31 plays an important role in regulating the cascade of invasion and metastasis. We further showed that miR-31 negatively regulated the *AFF1* mRNA level. The *AFF1* (which is also known as AF4) is one of the super elongation complex and was reported to be involved in the transcriptional elongation misregulation that resulted in leukemic pathogenesis [39]. In acute lymphoblastic leukaemia, the down-regulation of *AFF1* by miR-143 has induced apoptosis and suppressed leukemic cells growth. However, the role of *AFF1* solid tumor specifically in SEOC is not clear thus extensive work to elucidate the mechanisms of miR-31 and *AFF1* binding interaction in this cancer shall follow.

One of the caveats of this study is the absence of the metastases samples for our miRNA expression profiling. We believe that the inclusion of metastatic tissue samples as well as the ascites fluid could give us better insights on the roles of miRNAs in SEOC progression. Moreover, the qPCR panel used in this study consisted of frequently altered miRNAs in human cancer and therefore novel miRNAs that may be important to SEOC were not identified. The use of tissue array covering numerous tissue samples of different SEOC stages and metastases counterparts as well as normal ovarian for comparison are warranted to provide in-depth information on miR-200c and miR-31 expressions and localizations. Future studies examining miR-200c and miR-31 interaction with their putative targets should be tested using luciferase reporter assay and functional studies using *in vivo* models may provide a better understanding of SEOC pathogenesis.

## Conclusions

Our study revealed that 38 out of 85 tested miRNAs were aberrantly expressed in SEOC patients through qPCR expression profiling approach. We have demonstrated that miR-200c and miR-31 significantly affects cell proliferation, clonogenic potential, migration and invasion in SEOC cells. Our data provided insight into the identification of miRNA-based biomarker and suggested that modulation of these miRNAs with mimics or inhibitors could serve as a promising cancer gene therapy for metastasis inhibition in SEOC. The miRNA expression profile together with the generated putative miRNA targets and pathways offer a basis for future studies to understand the metastasis mechanisms of SEOC.

## Additional files

Additional file 1: Table S1. Cancer-related miRNAs included in the Cancer Focus microRNA Panel V1. (DOCX 13 kb) Additional file 2: Table S2. Complete list of the 85 miRNAs evaluated across all samples. (XLSX 50 kb) Additional file 3: Figure S1. The representative image of ISH showing the miR-200c blue chromogenic signal in the cytoplasmic region of a high-grade SEOC cancer epithelia and weak staining in the neighbouring stroma cells. Positive miR-200c staining was also noted in the nucleoli of SEOC cells. Image was captured at 200× magnifications. **Figure S2**. Expression of miR-31 in tissue and cell lines of serous ovarian cancer. (A) Expression of miR-31 in serous ovarian cancer compared to the normal ovarian tissue samples. (B) Expression of miR-31 in two serous ovarian cancer cell lines, CAOV3 and SKOV3 compared to the HOSE, the human normal ovarian surface epithelial cells. Data are presented as means ± standard deviation generated from triplicates. (****p* < 0.05). **Figure S3**. Detection of miRNA transfection efficiency in (A) CAOV3 and (B) SKOV3 cells. Twenty four hours after transfection with 150 nM 5’ fluorescein-labeled scrambled miRNA, the transfection efficiency was determined by flow cytometry. The P1 region represents the percentage of cells that were successfully transfected with 5’ fluorescein-labeled scrambled miRNA by Lipofectamine 2000. Mock transfection represents cells treated with Lipofectamine 2000 only. The results were analyzed with FACS Diva Version 6.1.3 software, which indicated that the miRNA transfection efficiency in CAOV3 and SKOV3 cells were approximately 60 % and 80 %, respectively. **Table S3**. Summary of the pathway enrichment analysis and putative target genes for miR-200c. **Table S4**. Summary of the pathway enrichment analysis and putative target genes for miR-31. (DOCX 2955 kb)
