# Supplementary material for: MicroRNA-200c and microRNA-31 regulate proliferation, colony formation, migration and invasion in serous ovarian cancer
Source: J Ovarian Res. 2015 Aug 12;8:56. doi: 10.1186/s13048-015-0186-7 (PMC4531514; doi:10.1186/s13048-015-0186-7)
Supplement: Additional file 1: Table S1. — Cancer-related miRNAs included in the Cancer Focus microRNA Panel V1. (DOCX 13 kb) [file 13048_2015_186_MOESM1_ESM.docx]

**Additional file 1**

**Table S1. Cancer-related miRNAs included in the Cancer Focus microRNA Panel V1**

| let-7a | let-7b | let-7c | let-7d | let-7e |
| --- | --- | --- | --- | --- |
| let-7f | let-7g | let-7i | miR-1 | miR-100 |
| miR-101 | miR-106a | miR-106b | miR-107 | miR-10a |
| miR-10b | miR-125b | miR-126 | miR-130a | miR-132 |
| miR-133a | miR-141 | miR-143 | miR-145 | miR-146a |
| miR-148a | miR-149* | miR-150 | miR-155 | miR-15a |
| miR-15b | miR-16 | miR-17 | miR-181a | miR-181b |
| miR-181d | miR-182 | miR-183 | miR-186 | miR-18a |
| miR-192 | miR-194 | miR-195 | miR-196a | miR-19a |
| miR-19b | miR-200a | miR-200b | miR-200c | miR-202 |
| miR-203 | miR-205 | miR-206 | miR-20a | miR-20b |
| miR-21 | miR-210 | miR-214 | miR-215 | miR-22 |
| miR-221 | miR-222 | miR-223 | miR-23a | miR-23b |
| miR-24 | miR-25 | miR-26a | miR-26b | miR-27a |
| miR-27b | miR-29a | miR-29b | miR-29c | miR-30b |
| miR-30c | miR-30d | miR-31 | miR-34a | miR-7 |
| miR-9 | miR-92a | miR-92b | miR-93 | miR-99a |
