# Supplementary material for: MicroRNA-200c and microRNA-31 regulate proliferation, colony formation, migration and invasion in serous ovarian cancer
Source: J Ovarian Res. 2015 Aug 12;8:56. doi: 10.1186/s13048-015-0186-7 (PMC4531514; doi:10.1186/s13048-015-0186-7)
Supplement: Additional file 3: Figure S1. — The representative image of ISH showing the miR-200c blue chromogenic signal in the cytoplasmic region of a high-grade SEOC cancer epithelia and weak staining in the neighbouring stroma cells. Positive miR-200c staining was also noted in the nucleoli of SEOC cells. Image was captured at 200× magnifications. Figure S2. Expression of miR-31 in tissue and cell lines of serous ovarian cancer. (A) Expression of miR-31 in serous ovarian cancer compared to the normal ovarian tissue samples. (B) Expression of miR-31 in two serous ovarian cancer cell lines, CAOV3 and SKOV3 compared to the HOSE, the human normal ovarian surface epithelial cells. Data are presented as means ± standard deviation generated from triplicates. (***p < 0.05). Figure S3. Detection of miRNA transfection efficiency in (A) CAOV3 and (B) SKOV3 cells. Twenty four hours after transfection with 150 nM 5’ fluorescein-labeled scrambled miRNA, the transfection efficiency was determined by flow cytometry. The P1 region represents the percentage of cells that were successfully transfected with 5’ fluorescein-labeled scrambled miRNA by Lipofectamine 2000. Mock transfection represents cells treated with Lipofectamine 2000 only. The results were analyzed with FACS Diva Version 6.1.3 software, which indicated that the miRNA transfection efficiency in CAOV3 and SKOV3 cells were approximately 60 % and 80 %, respectively. Table S3. Summary of the pathway enrichment analysis and putative target genes for miR-200c. Table S4. Summary of the pathway enrichment analysis and putative target genes for miR-31. (DOCX 2955 kb) [file 13048_2015_186_MOESM3_ESM.docx]

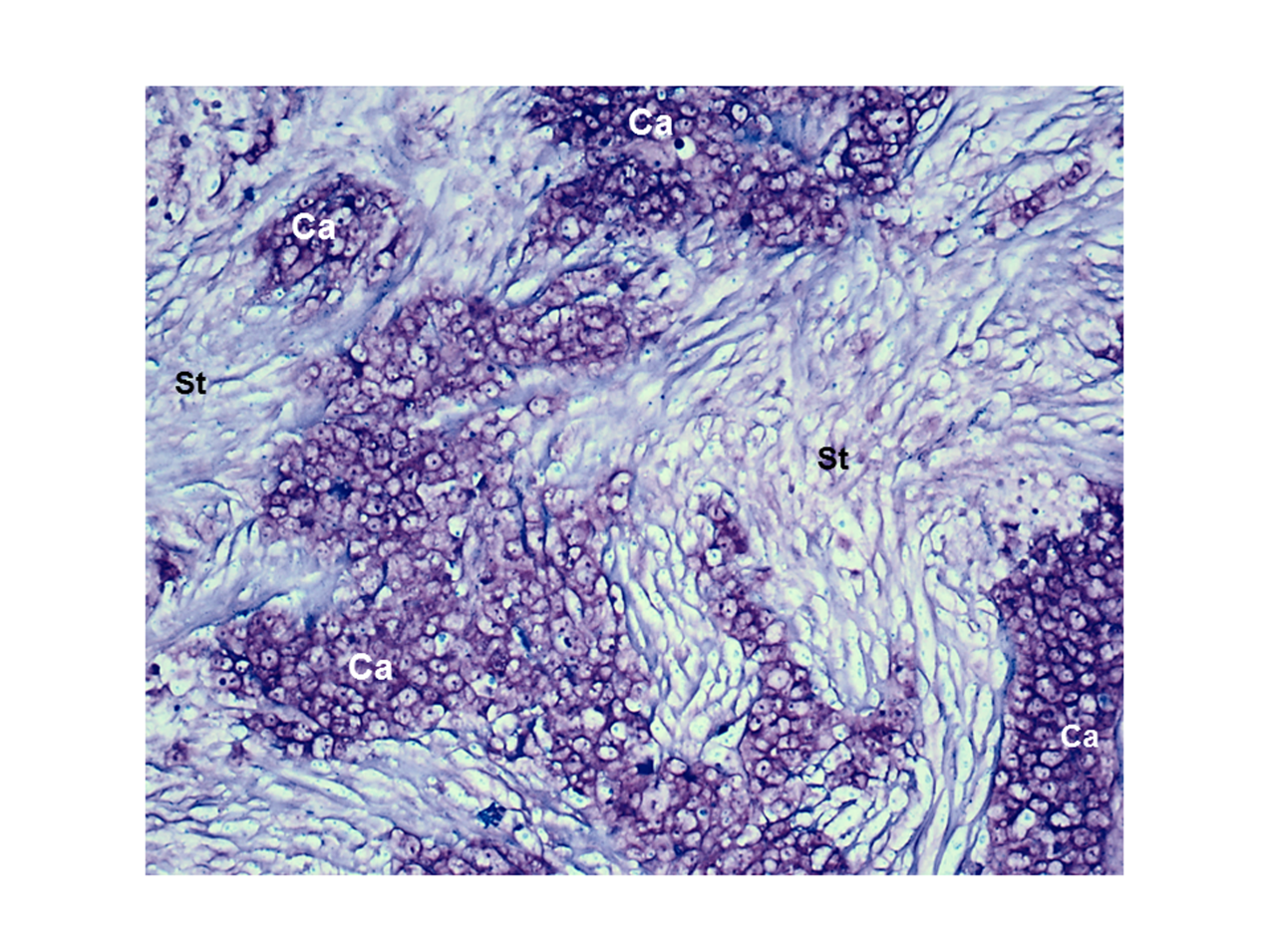


**Figure S1.** The representative image of ISH showing the miR-200c blue chromogenic signal in the cytoplasmic region of a high-grade SEOC cancer epithelia and weak staining in the neighbouring stroma cells. Positive miR-200c staining was also noted in the nucleoli of SEOC cells. Image was captured at 200× magnifications.


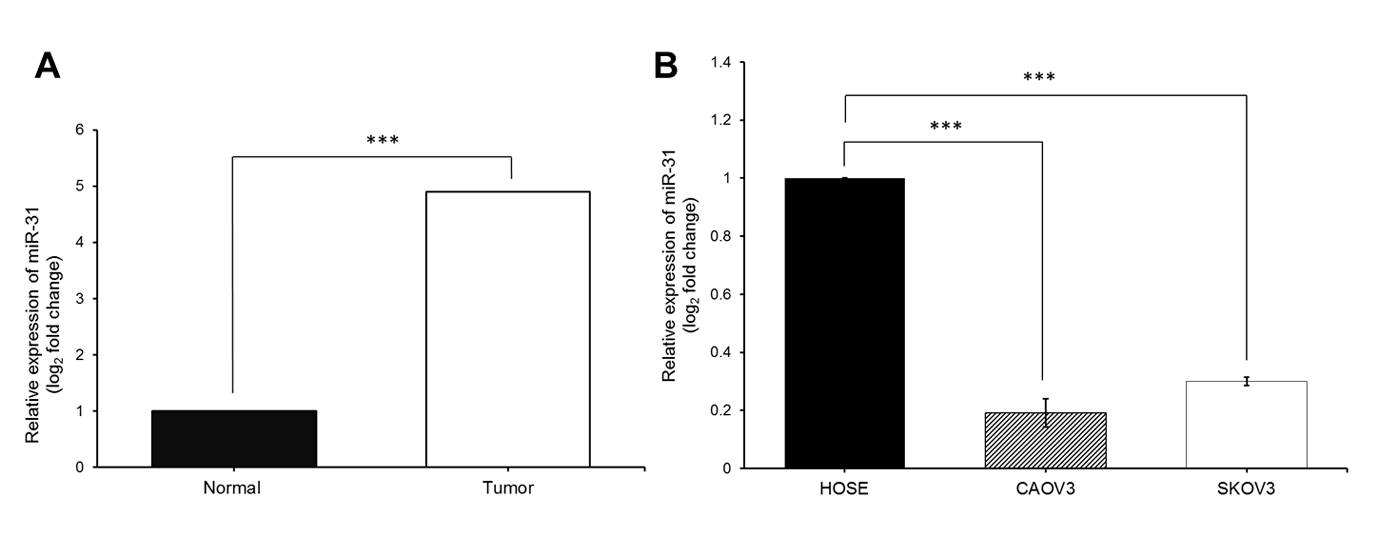


**Figure S2.** Expression of miR-31 in tissue and cell lines of serous ovarian cancer. (A) Expression of miR-31 in serous ovarian cancer compared to the normal ovarian tissue samples. (B) Expression of miR-31 in two serous ovarian cancer cell lines, CAOV3 and SKOV3 compared to the HOSE, the human normal ovarian surface epithelial cells. Data are presented as means ± standard deviation generated from triplicates. (***p<0.05).


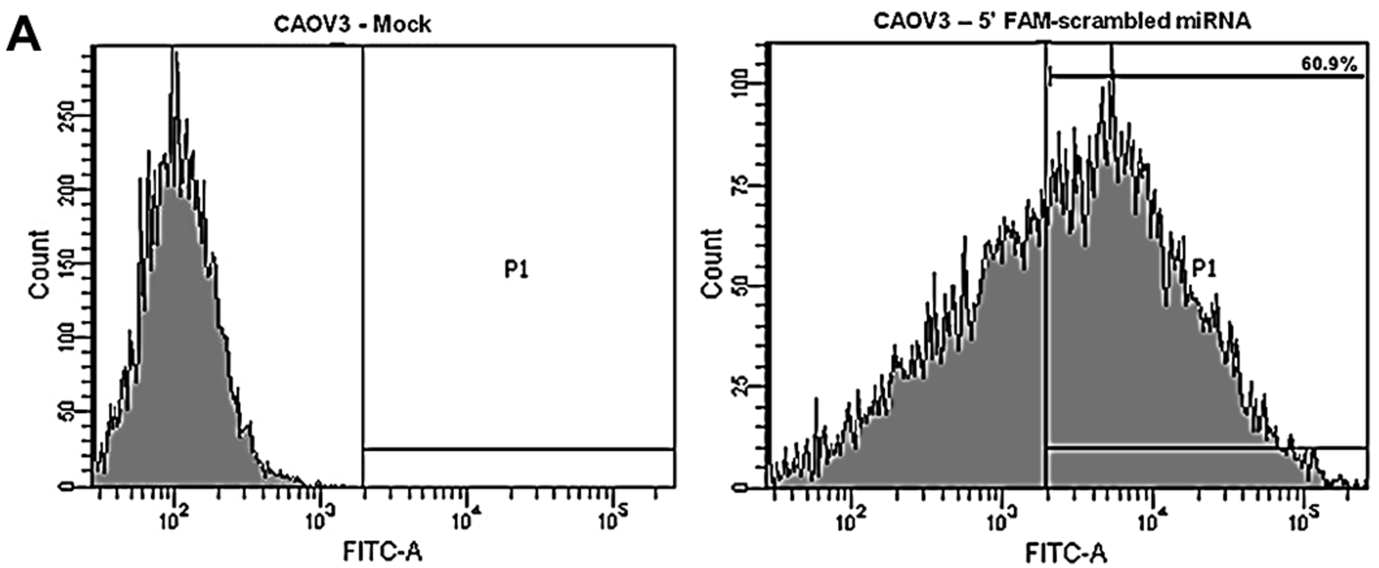


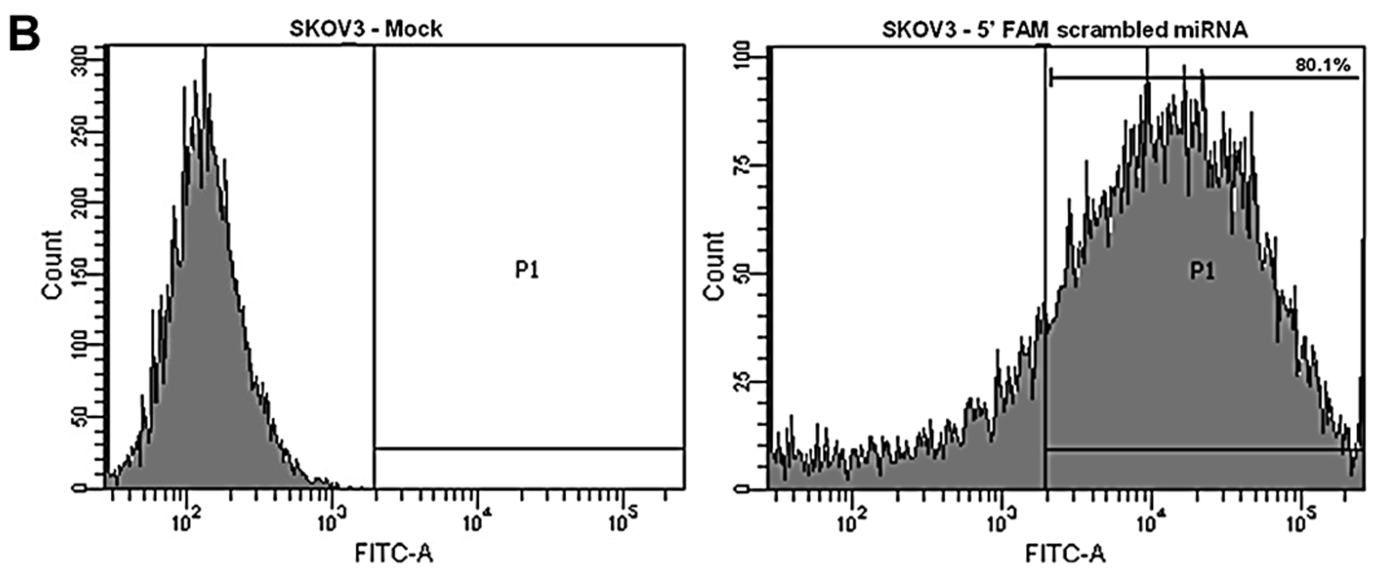


**Figure S3.** Detection of miRNA transfection efficiency in (**A**) CAOV3 and (**B**) SKOV3 cells. Twenty four hours after transfection with 150 nM 5’ fluorescein-labeled scrambled miRNA, the transfection efficiency was determined by flow cytometry. The P1 region represents the percentage of cells that were successfully transfected with 5’ fluorescein-labeled scrambled miRNA by Lipofectamine 2000. Mock transfection represents cells treated with Lipofectamine 2000 only. The results were analyzed with FACS Diva Version 6.1.3 software, which indicated that the miRNA transfection efficiency in CAOV3 and SKOV3 cells were approximately 60% and 80%, respectively.

**Table S3.** Summary of the pathway enrichment analysis and putative target genes for miR-200c.

| miRNA | Cancer-related pathway (KEGG) | Putative target genes |
| --- | --- | --- |
| miR-200c | Acute myeloid leukemia | AFF1, IKBKB, PIM2, MYC |
|  | Bladder cancer | MYC |
|  | Cell cycle | CDK2, CREBBP, YWHAG |
|  | Colorectal cancer | JUN, MYC |
|  | Cytokine-cytokine receptor | FLT4, CLCF1, KDR, IFNA2 |
|  | Endometrial cancer | MYC |
|  | ERBB signaling pathway | PLCG1, JUN, PAK7, MYC |
|  | Focal adhesion | JUN, KDR, PAK7, ROCK2, FYN, TLN1, DLC1 |
|  | Glioma | PLCG1 |
|  | Hedgehog signaling pathway | CSNK1G3, WNT16 |
|  | JAK-STAT signaling pathway | CREBBP, CLCF1, IFNA2, MYC |
|  | MAPK signaling pathway | CACNA2D4, IKBKB, DUSP1, FGF9, JUN, MAP3K1, MAPK7, CACNB2, RAPGEF2, MYC, MAP2K5, TRAF6 |
|  | mTOR signaling pathway | ULK2 |
|  | Notch signaling pathway | CREBB2, NCOR2, NOTCH1 |
|  | p53 signaling pathway | CDK2, SESN1, PMAIP1 |
|  | Pancreatic cancer | IKBKB |
|  | Prostate cancer | IKBKB, CDK2, CREBBP |
|  | TGF-β signaling pathway | CREBB2, PPP2R2C, NOG, ROCK2, MYC |
|  | VEGF signaling pathway | PLCG1, KDR |
|  | Wnt signaling pathway | WIF1, CREBBP, JUN, WNT16, PPP2R2C, ROCK2, MYC |

**Table S4.** Summary of the pathway enrichment analysis and putative target genes for miR-31.

| miRNA | Cancer-related pathway (KEGG) | Putative target genes |
| --- | --- | --- |
| miR-31 | Acute myeloid leukemia | KRAS, STAT5A, AFF1 |
|  | Bladder cancer | FGFR3, KRAS, VEGFB |
|  | Cell cycle | SMC1B, SFN, ANAPC11, YWHAE, CDC6 |
|  | Colorectal cancer | KRAS, DVL3, AXIN1, CASP9 |
|  | Cytokine-cytokine receptor | IL12RB2, PLEKHO2, CLCF1, IL1RAP, PPBP |
|  | ECM receptor interaction | FNDC3A, FNDC4, AGRN, SDC1 |
|  | Endometrial cancer | KRAS, AXIN1, ILK, CASP9 |
|  | ERBB signaling pathway | KRAS, CRK, HBEGF, SHC4, STAT5A, CAMK2G, PAK4 |
|  | Focal adhesion | CRK, VAV3, SHC4, PAK4, ILK, VEGFB |
|  | Glioma | KRAS, SHC4, CAMK2G |
|  | JAK-STAT signaling pathway | IL12RB2, SPRED1, CLCF1, STAT5A, SOCS1 |
|  | MAPK signaling pathway | FGFR3, KRAS, CRK, MAP2K1IP1, FGF9, PPP3CA, MAPK11, CACNB2, CACNB3, CACNA2D2, NTF3, MAP2K5 |
|  | mTOR signaling pathway | LYK5, VEGFB |
|  | Notch signaling pathway | DVL3 |
|  | p53 signaling pathway | SFN, DDB2, CASP9 |
|  | Pancreatic cancer | KRAS, VEGFB, CASP9 |
|  | PPAR signaling pathway | RXRB, ACADL, ILK |
|  | Prostate cancer | KRAS, CASP9 |
|  | TGF-β signaling pathway | PPP2R2A, SP1, ACVRL1, INHBC |
|  | VEGF signaling pathway | KRAS, NOS3, PPP3CA, MAPK11, CASP9 |
|  | Wnt signaling pathway | PPP2R2A, DVL3, PLCB1, PPP3CA, CAMK2G, AXIN1, CSNK2A2 |
